# Cell Damage at the Origin of Antiphospholipid Antibodies and Their Pathogenic Potential in Recurrent Pregnancy Loss

**DOI:** 10.1155/S1064744997000264

**Published:** 1997

**Authors:** Véronique Piroux, Valérie Eschwège, Jean-Marie Freyssinet

**Affiliations:** 1 Institut d'Hématologie et d'Immunologie Faculté de Médecine Université Louis Pasteur 4, rue Kirschleger Strasbourg 67085 France; 2 Unité 143 INSERM Hôpital de Bicêtre Le Kremlin-Bicêtre France

## Abstract

Antiphospholipid antibodies (APA) are associated with thrombosis, thrombocytopenia and fetal loss but they occur in a variety of diseases. Despite many efforts, a correlation between the specificity of particular subgroups of APA and particular clinical situations remains to be established. The antigens at the origin of APA remain to be identified. We discuss here the possible links between cell apoptosis or necrosis, leading to plasma membrane alterations, and the occurrence of APA in response to sustained stimulation. The pathogenic potential of APA is also considered with respect to recurrent pregnancy loss.


Infectious Diseases in Obstetrics and Gynecology 5:176–180 (1997)
© 1997 Wiley-Liss, Inc.

### Cell Damage at the Origin of Antiphospholipid Antibodies and Their Pathogenic Potential in Recurrent Pregnancy Loss

**Véronique Piroux, Valérie Eschwège, and Jean-Marie Freyssinet**

*Institut d'Hématologie et d'Immunologie, Faculté de Médecine, Université Louis Pasteur, Strasbourg (V.P., J.-M.F.); Unité 143 INSERM, Hôpital de Bicêtre, Le Kremlin-Bicêtre; France (V.E., J.-M.F.)*

ABSTRACT

**Antiphospholipid antibodies (APA) are associated with thrombosis, thrombocytopenia and fetal loss but they occur in a variety of diseases. Despite many efforts, a correlation between the specificity of particular subgroups of APA and particular clinical situations remains to be established. The antigens at the origin of APA remain to be identified. We discuss here the possible links between cell apoptosis or necrosis, leading to plasma membrane alterations, and the occurrence of APA in response to sustained stimulation. The pathogenic potential of APA is also considered with respect to recurrent pregnancy loss. Infec. Dis. Obstet. Gynecol. 5:176–180, 1997.**
© 1997 Wiley-Liss, Inc.

KEY WORDS

Apoptosis, necrosis, membrane phospholipid asymmetry, annexin V, procoagulant lipid exposure

### ROUTINE DETECTION OF ANTIPHOSPHOLIPID ANTIBODIES

Antiphospholipid antibodies (APA) are frequently detected in routine laboratory practice. Various targets are used in ELISA. However their reactivity is highly heterogeneous. The different phospholipids used are either negatively-charged, cardiolipin and phosphatidylserine, or neutral like phosphatidylethanolamine, or mixtures. Concerning the protein components of complex antigens, the importance of β2-glycoprotein I and prothrombin is now widely accepted. Some other phospholipid-binding proteins like kininogens, protein C, protein S or annexin V could also expose neoepitopes when bound to phospholipids.[1,2] Under certain *in vitro* conditions, the presence of phospholipid is not even required for APA binding to protein cofactors.[3] *In vivo,* it is certainly conceivable that phospholipids are needed to present the proteins. APA probably result from the reactivity of the immune system to membrane components that are normally sequestered in the absence of cell stimulation and/or to plasma proteins showing a certain affinity for such components when accessible. The different modes of phospholipid exposure combined with the binding of different proteins could explain the heterogeneity of APA.

### EXPOSURE OF SEQUESTERED PHOSPHOLIPIDS

Cardiolipin (diphosphatidylglycerol), phosphatidylserine and phosphatidylethanolamine are the most frequent phospholipids used as targets for APA. Phosphatidylserine and phosphatidylethanolamine are ubiquitous aminophospholipid components of eukaryotic cell plasma membranes. In

Correspondence to: J.-M. Freyssinet, Institut d'Hématologie et d'Immunologie, Faculté de Médecine, 4, rue Kirschleger, 67085 Strasbourg, France; e-mail: Jean-Marie.Freyssinet@hemato-ulp.u-strasbg.fr

Received 1 October 1997

Accepted 21 October 1997

resting cells, they are mostly restricted to the inner leaflet of the membrane.[4] The ability of phosphatidylethanolamine to adopt hexagonal phase organization could explain its immunogenicity, but the physiopathological conditions allowing this conformation are probably not frequent. Cardiolipin is mainly localized in the inner membrane of the mitochondria. A drastic lesion, due to mechanical stress[5] or necrosis, is necessary to expose this phospholipid. Apoptosis and necrosis, as well as inflammation and infection, are cell death pathways and clinical situations during which the loss of membrane asymmetry is observed. Resting platelets, monocytes, endothelial cells and red blood cells present an asymmetric distribution of the phospholipid constituents of their plasma membrane. Stimulated cells, apoptotic cells and bodies, shed microparticles and necrotic fragments present membrane rearrangements.

Aminophospholipids become exposed at the outer surface of the plasma membrane of stimulated cells and that of shed microparticles. The most frequent example is that of platelets,[4] but monocytes[6] and endothelial cells[7] are also able to elicit a similar membrane response. Once accessible to plasma vitamin K-dependent clotting factors, phosphatidylserine, in cooperation with phosphatidylethanolamine, acquires the ability to promote the assembly of the characteristic enzyme complexes of the coagulation cascade.[4] Different clinical situations are associated with increased circulating platelet microparticles.[8–13] One of them, idiopathic thrombocytopenic purpura, is further characterized by the three following observations: APA positivity is a common feature in patients with idiopathic thrombocytopenic purpura,[14] APA are able to bind to platelet microparticles in idiopathic thrombocytopenic purpura,[15] and platelet microparticles in idiopathic thrombocytopenic purpura are enriched in β2-glycoprotein I.[16] This is certainly suggestive of the generation of APA in response to an increase of platelet microparticles.

Inflammation is a common pathological situation in which increased aminophospholipid inside-out migration and microparticle shedding are likely to occur. Proinflammatory mediators, interleukin 1-β and tumor necrosis factor-α, induce procoagulant phospholipid exposure, increased thrombin generation and subsequent or concomitant cell-cell interactions. When combined, these effects result in a dramatic augmentation of the thrombotic risk. APA have been reported to inhibit procoagulant activity of activated platelets and platelet-derived microparticles.[17] Hence, two questions arise from these considerations: do APA occur in response to sustained exposure of thrombogenic phospholipid surfaces?, and the corollary: are APA first synthesized as 'protecting antibodies'?

The APA associated with infection have been claimed to be β2-glycoprotein I-independent antibodies and probably not related to thrombosis. Nevertheless, it is not an easy task to discriminate between these two different types of antibodies. In bacteria, different membrane components such as lipid A and cardiolipin are candidate targets for APA. Concerning parasites, phosphatidylserine expression at the surface of malaria-parasitized erythrocytes is now well described. More data are available regarding viruses. Membrane rearrangements occur during fusion and viral maturation of enveloped viruses. The envelope is a modified host cell plasma membrane.[18] In all cases of infection, complement-mediated cell lysis leads to inflammation and increased circulating cell fragments. The association between hepatitis C virus and APA has been studied in patients with hemophilia[19] and HIV infection.[20] Furthermore, the increased rate of thrombin generation in hepatitis C virus cirrhotic patients,[21] combined with the increased prevalence of anticardiolipin antibodies in chronic hepatitis C,[22] suggests an implication of this virus in the associated antiphospholipid syndrome.

Exposure of phosphatidylserine at the surface of apoptotic cells and derived fragments is an early feature of apoptosis.[23,24] Fadok et al. and other groups demonstrated that phosphatidylserine triggers recognition and removal by phagocyte.[25–27] The identity of phosphatidylserine receptor(s) remains to be established but β2-glycoprotein I could be involved, as it has been detected on rapidly-cleared liposomes.[28] Apoptotic cells and bodies have comparable procoagulant properties as activated platelets and shed microparticles which can be counteracted by purified immunoglobulin G from patients with APA syndrome. The procoagulant potential of apoptotic cells and derived fragments could lead to pathologic coagulation and their immunologic potential could be responsible for the generation of APA.[29] Hence, APA are expected to occur in diseases known to be associated

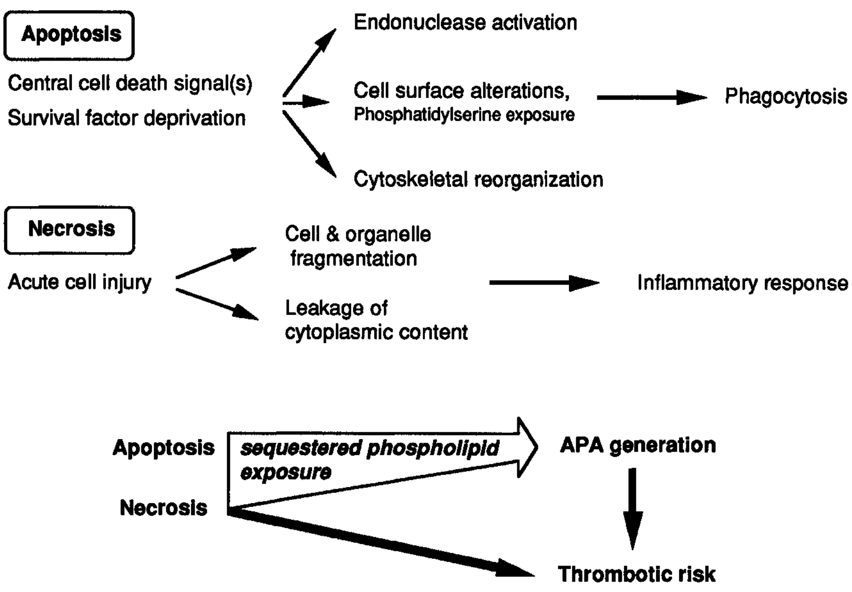

Fig. 1. The possible contribution of programmed cell death (apoptosis) and necrosis in the occurrence of antiphospholipid antibodies (APA). Phosphatidylserine and phosphatidylethanolamine are sequestered in the inner leaflet of the plasma membrane of unstimulated cells. Cardiolipin is mainly found in the inner membrane of mitochondria. Hence, an apoptotic process is expected to lead to phosphatidylserine and phosphatidylethanolamine externalization. In addition to these two lipids, cardiolipin becomes accessible after cell necrosis.

with increased apoptosis such as AIDS, myocardial infarction and stroke.[30] Another situation of increased apoptosis is alcoholic intoxication.[30] Chedid et al. found that the prevalence of APA increases dramatically in parallel with liver function impairment.[31]

Finally, it is important to emphasize the major differences between apoptosis and necrosis. Apoptosis is induced by central cell death signal(s) or survival factor deprivation and is characterized by endonuclease activation, surface alterations and cytoskeletal reorganization. The different cell surface alterations are as many triggers for removal by phagocytes and, except when the reticuloendothelial system is saturated, cell death and elimination occur rapidly without inflammation. At the opposite, necrosis is an acute cell injury accompanied by leakage of cytoplasmic content and by the disintegration of lysosomes and mitochondria which are responsible for an inflammatory response.[30] Various mechanisms including apoptosis and necrosis could be responsible for APA generation, but, because of the associated inflammatory response, necrosis could represent a true thrombotic risk. This does not rule out the possible pathogenicity of APA when synthesized in large excess (Figure 1).

### A POSSIBLE THROMBOGENIC MECHANISM AT THE ORIGIN OF PREGNANCY LOSS

Several pathogenic mechanisms have been proposed to explain the association of APA with an increased thrombotic risk,[32] but none regarding a possible interference of APA in pregnancy.

A recent contribution deserves particular attention regarding a possible mechanism of recurrent fetal loss.[33] These authors have evidenced the presence of annexin V on placental villi and confirmed their observation on cultured trophoblasts and endothelial cells. Owing to its strong affinity for phosphatidylserine, annexin V, a protein whose physiologic function remains to be established, exerts a potent *in vitro* anticoagulant effect. Its association with the outer leaflet of the plasma membrane of tissues fulfilling a barrier function may originate from the active membrane remodeling occurring in these cells. Aminophospholipid exposure and microvesicle shedding result in the development of procoagulant catalytic surfaces which have to be neutralized. Annexin V, also translocated during these membrane events, could precisely achieve such an anticoagulant control. The investigators have observed that annexin V levels are markedly reduced on placental villi from women presenting the antiphospholipid-antibody syndrome. In addition, they have shown that APA from three patients who experienced repeated fetal loss are able to displace annexin V from cultured trophoblasts or endothelial cells. The reduction of surface annexin V resulted in increased cell surface procoagulant potential. This provides a rationale for the recurrence of pregnancy loss.

### CONCLUSION—PERSPECTIVES

The antiphospholipid-antibody syndrome may reflect cell damage at the origin of the so-called APA, which in turn may acquire pathogenic potential depending on special circumstances. The cellular alterations may lead to a thrombogenic phenotype which has to be counteracted. Nevertheless, we are left with an immunologic problem. Solutions should be found in the light of the possible correlation(s) between the presence of particular subsets of APA and the occurrence of documented clinical manifestations. This implies that APA are better characterized at a molecular/gene level which should help to explore their specificity towards better defined antigens.

Because programmed cell death is of fundamental significance in embryogenesis and development,[30] a challenging demonstration would be that of an interference of APA in an apoptotic process resulting in pregnancy loss or fetal growth retardation.

### REFERENCES

1. Triplett DA: Protean clinical presentation of antiphospholipid-protein antibodies (APA). Thromb Haemost 74:329–337, 1995.
2. Galli M, Finazzi G, Barbui T: Antiphospholipid antibodies: predictive value of laboratory tests. Thromb Haemost 78:75–78, 1997.
3. Arvieux J, Darnige L, Hachulla E, et al.: Species specificity of anti-beta 2 glycoprotein I autoantibodies and its relevance to anticardiolipin antibody quantitation. Thromb Haemost 75:725–730, 1996.
4. Zwaal RFA, Schroit AJ: Physiopathologic implications of membrane phospholipid asymmetry in blood cells. Blood 89:1121–1132, 1997.
5. Antonopoulou S, Demopoulos C, Iatrou C: Blood cardiolipin in haemodialysis patients, its implication in the biological action of platelet-activating factor. Int J Biochem Cell Biol 28:43–51, 1996.
6. Satta N, Toti F, Feugeas O, et al.: Monocyte vesiculation is a possible mechanism for dissemination of membrane-associated procoagulant activities and adhesion molecules after stimulation by lipopolysaccharide. J Immunol 153:3245–3255, 1994.
7. Hamilton KK, Hattori R, Esmon CT, Sims PJ: Complement proteins C5b-9 induce vesiculation of the endothelial plasma membrane and expose catalytic surface for assembly of the prothrombinase enzyme complex. J Biol Chem 265:3809–3814, 1990.
8. George J, Pickett E, Saucerman S, et al.: Platelet surface glycoproteins. Studies on resting platelets and platelet membrane microparticles in normal subjects, and observations in patients during adult respiratory distress syndrome and cardiac surgery. J Clin Invest 78:340–348, 1986.
9. Abrams C, Shattil SJ: Immunological detection of activated platelets in clinical disorders. Thromb Haemost 65:467–473, 1991.
10. Jy W, Horstman LL, Arse M, Ahn YS: Clinical significance of platelet microparticules in autoimmune thrombocytopenias. J Lab Clin Med 119:334–345, 1992.
11. Holme PA, Solum NO, Brosstad F, Roger M, Abdelnoor M: Demonstration of platelet-derived microvesicles in blood from patients with activated coagulation and fibrinolysis using a filtration technique and Western blotting. Thromb Haemost 72:666–671, 1994.
12. Warkentin TE, Hayward CPM, Boschkov LK, et al.: Sera from patients with heparin-induced thrombocytopenia generate platelet-derived microparticles with procoagulant activity: an explanation for the thrombotic complications of heparin-induced thrombocytopenia. Blood 84:3691–3699, 1994.
13. Galli M, Grassi A, Barbui T: Platelet-derived microvesicles in thrombotic thrombocytopenic purpura and hemolytic uremic syndrome. Thromb Haemost 75:427–431, 1996.
14. Stasi R, Stipa E, Masi M, et al.: Prevalence and clinical significance of elevated antiphospholipid antibodies in patients with idiopathic thrombocytopenic purpura. Blood 84:4203–4208, 1994.
15. Nomura S, Yanabu M, Fukuroi T, et al.: Antiphospholipid antibodies bind to platelet microparticles in idiopathic (autoimmune) thrombocytopenic purpura. Ann Hematol 65:46–49, 1992.
16. Nomura S, Yanabu M, Miyake T, et al.: Relationship of microparticles with beta(2)-glycoprotein I and P-selectin positivity to anticardiolipin antibodies in immune thrombocytopenic purpura. Ann Hematol 70:25–30, 1995.
17. Galli M, Bevers EM, Comfurius P, Barbui T, Zwaal RFA: Effect of antiphospholipid antibodies on procoagulant activity of activated platelets and platelet-derived microvesicles. Br J Haematol 83:466–472, 1993.
18. Aloia R, Tian H, Jensen F: Lipid composition and fluidity of the human immunodeficiency virus envelope and host cell plasma membranes. Proc Natl Acad Sci USA 90:5181–5185, 1993.
19. Al-Saeed A, Makris M, Malia RG, Preston FE, Greaves M: The development of antiphospholipid antibodies in haemophilia is linked to infection with hepatitis C. Br J Haematol 88:845–848, 1994.

20. Gotoh M, Matsuda J: Human immunodeficiency virus rather than hepatitis C virus infection is relevant to the development of an anti-cardiolipin antibody. Am J Hematol 50:220–222, 1995.
21. Violi F, Ferro D, Basili S, et al.: Increased rate of thrombin generation in hepatitis C virus cirrhotic patients. Relationship to venous thrombosis. J Invest Med 43: 550–554, 1995.
22. Prieto J, Yuste JR, Beloqui O, et al.: Anticardiolipin antibodies in chronic hepatitis C: Implication of hepatitis C virus as the cause of the antiphospholipid syndrome. Hepatology 23:199–204, 1996.
23. Martin SJ, Reutelingsperger CPM, McGahon AJ, et al.: Early redistribution of plasma membrane phosphatidylserine is a general feature of apoptosis regardless of the initiating stimulus: Inhibition by overexpression of Bcl-2 and Abl. J Exp Med 182:1545–1556, 1995.
24. Aupeix K, Hugel B., Martin T, et al.: The significance of shed membrane particles during programmed cell death in vitro, and in vivo, in HIV-1 infection. J Clin Invest 99:1546–1554, 1997.
25. Fadok V, Voelker DR, Campbell PA, Cohen JJ, Bratton DL, Henson PM: Exposure of phosphatidylserine on the surface of apoptotic lymphocytes triggers specific recognition and removal by macrophages. J Immunol 148:2207–2216, 1992.
26. Bennett MR, Gibson DF, Schwartz SM, Tait JF: Binding and phagocytosis of apoptotic vascular smooth muscle cells is mediated in part by exposure of phosphatidylserine. Circ Res 77:1136–1142, 1995.
27. Verhoven B, Schlegel RA, Williamson P: Mechanisms of phosphatidylserine exposure, a phagocyte recognition signal, on apoptotic T lymphocytes. J Exp Med 182: 1597–1601, 1995.
28. Chonn A, Semple SC, Cullis PR: β2-Glycoprotein I is a major protein associated with very rapidly cleared liposomes in vivo, suggesting a significant role in the immune clearance of "non-self" particles. J Biol Chem 270:25845–25849, 1995.
29. Casciola-Rosen L, Rosen A, Petri M, Schlissel M: Surface blebs on apoptotic cells are sites of enhanced procoagulant activity: Implications for coagulation events and antigenic spread in systemic lupus erythematosus. Proc Natl Acad Sci USA 93:1624–1629, 1996.
30. Thompson CB: Apoptosis in the pathogenesis and treatment of disease. Science 267:1456–1462, 1995.
31. Chedid A, Chadalawada KR, Morgan TR, et al.: Phospholipid antibodies in alcoholic liver disease. Hepatology 20:1465–1471, 1994.
32. Santoro SA: Antiphospholipid antibodies and thrombotic predisposition: Underlying pathogenetic mechanisms. Blood 83:2389–2391, 1994.
33. Rand JH, Wu XX, Andree HAM, et al.: Pregnancy loss in the antiphospholipid-antibody syndrome—A possible thrombogenic mechanism. N Engl J Med 337:154–160, 1997.
